# Partial genetic characterization of West Nile virus strains, New York State, 2000.

**DOI:** 10.3201/eid0704.010408

**Published:** 2001

**Authors:** G D Ebel, A P Dupuis, K Ngo, D Nicholas, E Kauffman, S A Jones, D Young, J Maffei, P Y Shi, K Bernard, L D Kramer

**Affiliations:** Arbovirus Laboratories, New York State Department of Health, 5668 State Farm Road, Slingerlands, NY 12159, USA. ebel@wadsworth.org

## Abstract

We analyzed nucleotide sequences from the envelope gene of 11 West Nile (WN) virus strains collected in New York State during the 2000 transmission season to determine whether they differed genetically from each other and from the initial strain isolated in 1999. The complete envelope genes of these strains were amplified by reverse transcription-polymerase chain reaction. The resulting sequences were aligned, the genetic distances were computed, and a phylogenetic tree was constructed. Ten (0.7%) of 1,503 positions in the envelope gene were polymorphic in one or more sequences. The genetic distances were 0.003 or less. WN virus strains circulating in 2000 were homogeneous with respect to one another and to a strain isolated in 1999.


### Partial Genetic Characterization of West Nile Virus Strains, New York State, 2000

**Gregory D. Ebel, Alan P. Dupuis II, Kiet Ngo, David Nicholas, Elizabeth Kauffman, Susan A. Jones, Donna Young, Joseph Maffei, Pei-Yong Shi, Kristen Bernard, and Laura D. Kramer**
New York State Department of Health, Slingerlands, New York, USA

We analyzed nucleotide sequences from the envelope gene of 11 West Nile (WN) virus strains collected in New York State during the 2000 transmission season to determine whether they differed genetically from each other and from the initial strain isolated in 1999. The complete envelope genes of these strains were amplified by reverse transcription-polymerase chain reaction. The resulting sequences were aligned, the genetic distances were computed, and a phylogenetic tree was constructed. Ten (0.7%) of 1,503 positions in the envelope gene were polymorphic in one or more sequences. The genetic distances were 0.003 or less. WN virus strains circulating in 2000 were homogeneous with respect to one another and to a strain isolated in 1999.

The first outbreak of West Nile (WN) virus infection in North America (1) was apparently the result of single introduction and subsequent amplification of WN virus among *Culex pipiens* mosquitoes and their avian hosts (2-4). Human disease was accompanied by an epizootic in which high death rates from severe meningoencephalitis and myocarditis were reported in some avian hosts, notably American Crows *(Corvus brachyrhynchos)* (5). RNA virus populations are subject to high mutation rates and may evolve rapidly under certain conditions (6-8). To determine whether WN virus genotypes circulating in New York during the 2000 transmission season differed from those isolated there in 1999, WN virus strains were collected from mosquito pools and dead vertebrates, the complete nucleotide sequences of the envelope genes were determined, and the sequences of these strains were compared with one another and with a strain isolated during 1999.

### Materials and Methods

WN virus was isolated from pools of infected mosquitoes collected throughout New York State and from vertebrate tissues submitted by the N.Y. State Wildlife Pathology Unit. Mosquitoes were collected overnight in standard miniature light traps or gravid traps, and they were pooled and sent to the New York State Arbovirus Laboratories. Pools of mosquitoes and vertebrate tissues were homogenized in 2 mL of mosquito diluent (20% heat-inactivated fetal bovine serum [FBS] in Dulbecco's phosphate-buffered saline plus 50 µg/mL penicillin/streptomycin, 50 µg gentamicin, and 2.5 µg/mL fungizone) or 350 µL lysis buffer, respectively, by using an SPEX mixer-mill (Spex CertiPrep, Metuchen, NJ) and glass beads; 500 µL of the resulting suspension was transferred to 1.5-mL microcentrifuge tubes and centrifuged at 16,000 RCF for 10 min; 100 µL of the clarified solution was applied to confluent monolayers of African Green Monkey Kidney (Vero) cells in T-25 flasks, and virus was allowed to adsorb for 1 hr at 37°C, 5% $CO_2$. After adsorption, 5 mL of minimum essential medium (MEM) containing 2% FBS and antibiotics (as above) was applied to the cells, and they were returned to the incubator. Cultures were checked for signs of cytopathic effect (CPE) daily. When >50% of cells in a culture flask displayed CPE, the culture was harvested, and clarified aliquots of the culture media supernatant were supplemented with FBS (20% of final volume) and stored in 1.5-mL cryovials at -80°C until further use.

Virus stocks were passed by applying 100 µL of the initial culture supernatant to a second confluent monolayer of Vero cells, as above. When CPE was evident in >50% of the cells in the culture, the cells were scraped from the flask and centrifuged with the media in 15-mL conical tubes at 3,000 x *g* for 20 minutes. RNA was extracted from the resulting cell pellet by using RNeasy columns (Qiagen, Valencia, CA) as directed by the manufacturer. The complete envelope sequences were amplified by reverse transcription-polymerase chain reaction (RT-PCR) with primers (Forward [5-CATCGAATTCGTTACCCTCTCTAACTTC-CAAGGGAAGGTG-3] Reverse [5-GTATGGATCCTGATGCTC-CAGTCTGGAAACTGATCGTA-3]) designed to amplify the genomic sequence covering the coding region of the complete prM/M, E, and the N-terminal NS1. These primers also contain engineered restriction sites for use in other experiments that will be described elsewhere. Reaction products were electrophoretically separated on 2% agarose gel, and bands of the predicted size were excised and purified by using the Qiaquick gel extraction kit (Qiagen, Valencia, CA). Purified DNA fragments were sequenced on an ABI PRISM 377XL automated DNA sequencer (Applied Biosystems, CA) with six forward and six reverse primers (Table 1).

Two of the vertebrate strains (3282 and 3356) were processed differently. RT-PCR was conducted directly on RNA isolated from infected tissues. The primers used for the

Address for correspondence: Gregory Ebel, Arbovirus Laboratories, 5668 State Farm Road, Slingerlands, NY 12159, USA; fax: 518-869-4530; e-mail: ebel@wadsworth.org

Table 1. Primers used in West Nile virus sequencing

| Primer | Sequence |
|---|---|
| WNSE1F | CTC TCT AAC TTC CAA GGG AAG |
| WNSE2F | CAC TCT AGC GAA CAA GAA GG |
| WNSE3F | TCT CCA CCA AAG CTG CGT GC |
| WNSE4F | TAC TAC GTG ATG ACT GTT GGA A |
| WNSE5F | CCT TGC AAA GTT CCT ATC TC |
| WNSE6F | TCC TGT TGT GGA TGG GCA TC |
| WNSE1R | TGT CTT CTG GAT CAT TAC CAG C |
| WNSE2R | GCC ACC AGG GCA TAT CCA GG |
| WNSE3R | TTC AAG ATG GTT CTT CCT ATT GC |
| WNSE4R | GGA ATG GCT CCA GCC AAA GC |
| WNSE5R | TGT TCT CCT CTG CCC ACC AC |
| WNSE6R | TCC ATC CAA GCC TCC ACA TC |

amplification and sequencing steps will be described elsewhere (Lanciotti et al., manuscript in preparation).

Sequences were aligned with a WN virus strain collected in 1999 (GenBank Accession #AF260967) and a distantly related St. Louis encephalitis virus sequence (AF205490) by using the clustal method on the DNAStar software package. Initial analysis was done by the distance method using MEGA (9). Evolutionary distances were computed by the Kimura 2 parameter method including both transitions and transversions. Distance trees were constructed by the neighbor-joining method, and their robustness was estimated by performing 500 bootstrap replicates.

### Results

WN virus strains from diverse locations, times, and host types were assembled for this study (Table 2). The strains were isolated from avian and mosquito hosts collected from midsummer through autumn at the epicenter and at the periphery of the 2000 epizootic. Strains were thus a representative sample of WN virus circulating in New York during 2000.

Nucleotide substitutions occurred at 10 (0.7%) of the 1,503 positions in the envelope gene (Table 3). Of these substitutions, all were transitions, two (0.4%) of which resulted in amino acid changes. The C to U substitution at position 2321 (position numbers refer to Lanciotti et al. [1]) results in a serine to leucine change in envelope amino acid number 452, and the A to G substitution at position 2386 results in an isoleucine to valine change in envelope amino acid position 474. The mean pairwise Kimura 2-parameter distances between the isolates were 0.003 or less. The phylogenetic tree of the nucleotide sequences studied showed similarly minimal distances between the isolates, with low bootstrap confidence values at the nodes separating the branches. WN virus strains circulating in New York State during the 2000 transmission season were relatively homogeneous at both the nucleotide and amino acid levels.

### Conclusion

These data represent the first population study of WN virus in North America since its introduction in 1999. The envelope sequences of these virus strains establish a baseline sequence dataset against which strains isolated during future transmission seasons may be compared.

Only the envelope sequences were studied, which were analyzed by using distance matrices and neighbor-joining methods. Although complete genome sequences may have provided additional information, short sequence fragments have often been used in population studies of arboviruses (10-13). Additional criteria (maximum parsimony and maximum likelihood) may have provided corroboration for the close relationships observed; however, the sequences are so similar and the nodes on the neighbor-joining tree so poorly supported that additional analyses seemed unwarranted. Given the close relationship of the strains, it is unlikely that additional nucleotide data or analytic methods would have greatly enhanced our understanding of WN virus population structure in this hemisphere.

Mosquito- and vertebrate-derived sequences appeared to be distributed randomly in the phylogenetic tree. Date of isolation of the strains was similarly unimportant in the clustering of sequences. Additionally, passage history seemed not to affect the gene sequences. RT-PCR amplification directly from infected mosquito pools often failed or produced amplicons that did not conform to size expectations, necessitating Vero cell passage of many strains before amplification. The two sequences obtained from RNA extracts of infected tissue without Vero passage were not different from those passed through these cells twice. The WN virus sequences in this study were homogeneous with respect to passage history, host, and time.

A single nucleotide substitution, a C to U change at position 1974, was present in four of five strains isolated from Staten Island but not from other locations. This mutation caused these strains (3000017, 3100352, 3100365, and 3356) to cluster in the phylogenetic tree, but bootstrap confidence in this clustering pattern, as for all the relationships displayed (Figure), was low. The utility of this particular substitution for molecular epidemiologic studies of WN virus in North

Table 2. Characteristics of strains studied

| Strain | Collection date | County/borough | Site/town | Source | Passage history* | GenBank Accession No. |
|---|---|---|---|---|---|---|
| 3000017 | Jul-2000 | Staten Island | Richmond | *Cx. pipiens* | V2 | AF346309 |
| 3000259 | Jul-2000 | Suffolk | Calhoun | *Cx. pip/restuans* | V2 | AF346316 |
| 3000548 | Jul-2000 | Queens | Country Farm Museum | *Cx. pip/res* | V2 | AF346311 |
| 3000622 | Jul-2000 | Westchester | Twin Lake Stable | *Cx. pip/res* | V2 | AF346313 |
| 3100271 | Jul-2000 | Rockland | Unknown | *Cx. pip/res* | V2 | AF346312 |
| 3100352 | Jul-2000 | Staten Island | Saw Mill Marsh | *Cx. salinarius* | V2 | AF346314 |
| 3100365 | Jul-2000 | Staten Island | Fresh Kills Landfill | *Cx. pipiens* | V2 | AF346310 |
| 842 | Jul-2000 | Staten Island | Amboy Rd. | American Crow | V2 | AF346317 |
| 2741 | Sep-2000 | Albany | SUNY | American Crow | V2 | AF346315 |
| 3282 | Oct-2000 | Oswego | New Haven | Ruffed Grouse | P | AF346319 |
| 3356 | Oct-2000 | Staten Island | Mariner's Harbor | American Crow | P | AF346318 |

*V2=Two vero passages, P=primary RNA tissue extract

Table 3. Nucleotide substitutions in strains studied

| Strain | Position | | | | | | | | | |
|---|---|---|---|---|---|---|---|---|---|---|
| | 1285 | 1332 | 1449 | 1899 | 1974 | 2280 | 2321 | 2359 | 2386 | 2424 |
| NY99-EQHS | C | U | C | U | C | U | C | C | A | C |
| 3000017 | - | - | - | - | U | C | - | - | - | U |
| 3000259 | - | - | - | - | - | - | - | - | - | - |
| 3000548 | - | C | - | - | - | - | - | - | - | - |
| 3000622 | - | - | U | - | - | - | - | - | - | - |
| 3100271 | - | - | - | - | - | - | - | - | - | - |
| 3100352 | - | - | - | C | U | - | - | - | - | - |
| 3100365 | - | - | - | - | U | - | - | - | - | - |
| 842 | - | - | - | - | - | - | - | U | G | - |
| 2741 | - | - | - | - | - | - | - | - | - | - |
| 3282 | U | - | - | - | - | - | - | - | - | - |
| 3356 | U | - | - | - | U | - | U | - | - | - |

Position numbers correspond to Lanciotti et al.(1).

America is difficult to ascertain, but in principle, findings of this type may provide useful information in determining the mode or modes of spread of particular WN virus strains in North America.

The envelope sequences studied are highly conserved. RNA viruses are well known to exist as quasispecies, composed of a swarm of competing viral genotypes (14,15). This mode of existence, because of the lack of proofreading and mismatch-repair mechanisms of most viral encoded RNA-dependent RNA polymerases (16), may allow rapid evolution under certain circumstances. Dengue virus, another mosquito-borne flavivirus, is thought to have diversified as the viral population expanded with human and mosquito populations (17). WN virus, having entered a naïve ecosystem and vastly expanded its range, may evolve similarly. Many arboviruses, however, are remarkably conserved across time and space, implying stringent constraints on viral structural proteins and replicative machinery (18). Fitness of vesicular stomatitis virus, an animal RNA virus, has been shown to drop precipitously as the virus passed through a series of population bottlenecks (19). Whether WN virus will follow a pattern of diversification or conservation is unclear. The viruses in this study are likely the result of a single introduction of WN virus, primary expansion during 1999, overwintering, and secondary expansion during the 2000 transmission season. Determining the genetic structure of WN virus populations in subsequent transmission seasons may advance our understanding of WN virus perpetuation, selection, and evolution.

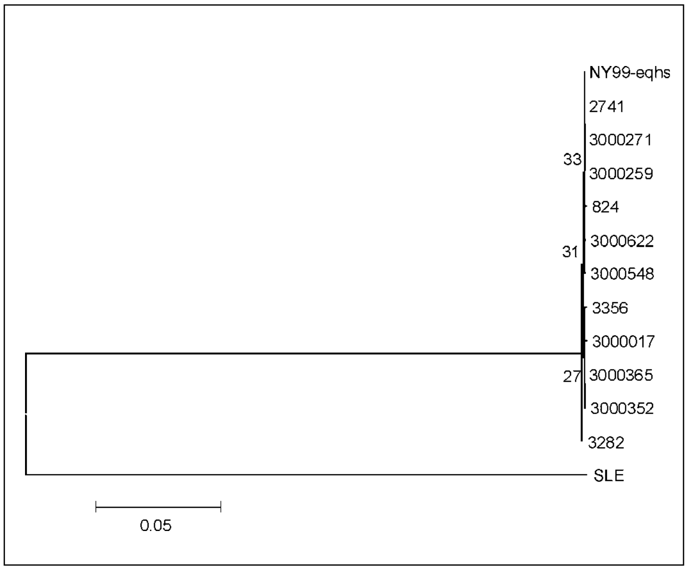

Figure. Phylogenetic relationships among West Nile virus strains collected in 2000. This tree is based on the 1503-bp envelope gene. Distance analysis based on Kimura 2-parameter distance including both transitions and transversions. Numbers at the nodes are bootstrap confidence estimates based on 500 replicates.

### Acknowledgments

We thank Robert Lanciotti and Amy Kerst for supporting the sequencing of 3282 and 3356; Harry Taber and the Wadsworth Center Molecular Genetics Core for institutional support at the Wadsworth Center; the Wadsworth Center Molecular Genetics Core; and the New York State Department of Health Division of Epidemiology, particularly Dennis White and Millicent Eidson, for guiding and supporting collection of the mosquito pools and vertebrates from which WN virus strains were isolated and for thoughtful comments on this manuscript.

Dr. Ebel is a research scientist in the Arbovirus Laboratories of the Wadsworth Center, New York State Department of Health. His research focuses on the population structure, biology, and molecular evolution of arthropod-borne zoonoses.

### References

1. Lanciotti RS, Roehrig JT, Deubel V, Smith J, Parker M, Steele K. et al. Origin of the West Nile virus responsible for an outbreak of encephalitis in the northeastern United States. Science 1999;286:2333-7.
2. Baqar S, Hayes CG, Murphy JR, Watts DM. Vertical transmission of West Nile virus by *Culex* and *Aedes* species mosquitoes. Am J Trop Med Hyg 1993;48:757-62.
3. Rappole JH, Derrickson SR, Hubalek Z. Migratory birds and spread of West Nile virus in the Western Hemisphere. Emerg Infect Dis 2000;6:319-28.
4. Komar N. West Nile viral encephalitis. Rev Sci Tech 2000;19:166-76.
5. Steele KE, Linn MJ, Schoepp RJ, Komar N, Geisbert TW, Manduca RM, et al. Pathology of fatal West Nile virus infections in native and exotic birds during the 1999 outbreak in New York City, New York. Vet Pathol 2000;37:208-24.
6. Holland JJ, Spindler K, Horodyski F, Grabau E, Nichol S, VandePol S. Rapid evolution of RNA genomes. Science 1982;215:1577-85.
7. Steinhauer DA, Holland JJ. Rapid evolution of RNA viruses. Annu Rev Microbiol 1987;41:409-33.

8. Garmendia AE, Van Kruiningen HJ, French RA, Anderson JF, Andreadis TG, Kumar A, et al. Recovery and identification of West Nile virus from a hawk in winter. J Clin Microbiol 2000;38:3110-1.
9. Kumar S, Tamura K, Nei M. MEGA: molecular evolutionary genetics analysis. University Park (PA): Pennsylvania State University Press; 1993.
10. Kramer LD, Presser SB, Hardy JL, Jackson AO. Genotypic and phenotypic variation of selected Saint Louis encephalitis viral strains isolated in California. Am J Trop Med Hyg 1997;57:222-9.
11. Harris E, Roberts TG, Smith L, Selle J, Kramer LD, Valle S, et al. Typing of dengue viruses in clinical specimens and mosquitoes by single-tube multiplex reverse transcriptase PCR. J Clin Microbiol 1998;36:2634-9.
12. Powers AM, Oberste MS, Brault AC, Rico-Hesse R, Schmura SM, Smith JF, et al. Repeated emergence of epidemic/epizootic Venezuelan equine encephalitis from a single genotype of enzootic subtype ID virus. J Virol 1997;71:6697-705.
13. Ebel GD, Foppa I, Spielman A, Telford SR. A focus of deer tick virus transmission in the northcentral United States. Emerg Infect Dis 1999;5:570-4.
14. Duarte EA, Novella IS, Weaver SC, Domingo E, Wain-Hobson S, Clarke DK, et al. RNA virus quasispecies: significance for viral disease and epidemiology. Infect Agents Dis 1994;3:201-14.
15. Holland JJ, de la Torre JC, Steinhauer DA. RNA virus populations as quasispecies. Curr Top Microbiol Immunol 1992;176:1-20.
16. Steinhauer DA, Domingo E, Holland JJ. Lack of evidence for proofreading mechanisms associated with an RNA virus polymerase. Gene 1992;122:281-8.
17. Zanotto PMDA, Gould EA, Gao GF, Harvey PH, Holmes EC. Population dynamics of flaviviruses revealed by molecular phylogenies. Proc Natl Acad Sci U S A 1996;93:548-53.
18. Pisano MR, Tolou H. The topotype notion and the quasispecies concept: The yellow fever virus as example. Travaux Scientifiques des Chercheurs du Service de Sante des Armees 1996;69-70.
19. Duarte E, Clarke D, Moya A, Domingo E, Holland J. Rapid fitness losses in mammalian RNA virus clones due to Mueller's ratchet. Proc Natl Acad Sci U S A 1992;89:6015-9.
